# Graphene Family Nanomaterials (GFN)-TiO_2_ for the Photocatalytic Removal of Water and Air Pollutants: Synthesis, Characterization, and Applications

**DOI:** 10.3390/nano11123195

**Published:** 2021-11-25

**Authors:** Chih-Hsien Lin, Wei-Hsiang Chen

**Affiliations:** 1 Institute of Environmental Engineering, National Sun Yat-sen University, Kaohsiung 804, Taiwan; sophia226@mail.nsysu.edu.tw; 2 Aerosol Science and Research Center, National Sun Yat-sen University, Kaohsiung 804, Taiwan; 3 Department of Public Health, Kaohsiung Medical University, Kaohsiung 807, Taiwan

**Keywords:** TiO_2_, graphene family nanomaterials (GFN), synthesis, surface characterization, photocatalytic removal, air and water pollutants

## Abstract

Given the industrial revolutions and resource scarcity, the development of green technologies which aims to conserve resources and reduce the negative impacts of technology on the environment has become a critical issue of concern. One example is heterogeneous photocatalytic degradation. Titanium dioxide (TiO_2_) has been intensively researched given its low toxicity and photocatalytic effects under ultraviolet (UV) light irradiation. The advantages conferred by the physical and electrochemical properties of graphene family nanomaterials (GFN) have contributed to the combination of GFN and TiO_2_ as well as the current variety of GFN-TiO_2_ catalysts that have exhibited improved characteristics such as greater electron transfer and narrower bandgaps for more potential applications, including those under visible light irradiation. In this review, points of view on the intrinsic properties of TiO_2_, GFNs (pristine graphene, graphene oxide (GO), reduced GO, and graphene quantum dots (GQDs)), and GFN-TiO_2_ are presented. This review also explains practical synthesis techniques along with perspective characteristics of these TiO_2_- and/or graphene-based materials. The enhancement of the photocatalytic activity by using GFN-TiO_2_ and its improved photocatalytic reactions for the treatment of organic, inorganic, and biological pollutants in water and air phases are reported. It is expected that this review can provide insights into the key to optimizing the photocatalytic activity of GFN-TiO_2_ and possible directions for future development in these fields.

## 1. Introduction

**Figure 1:**
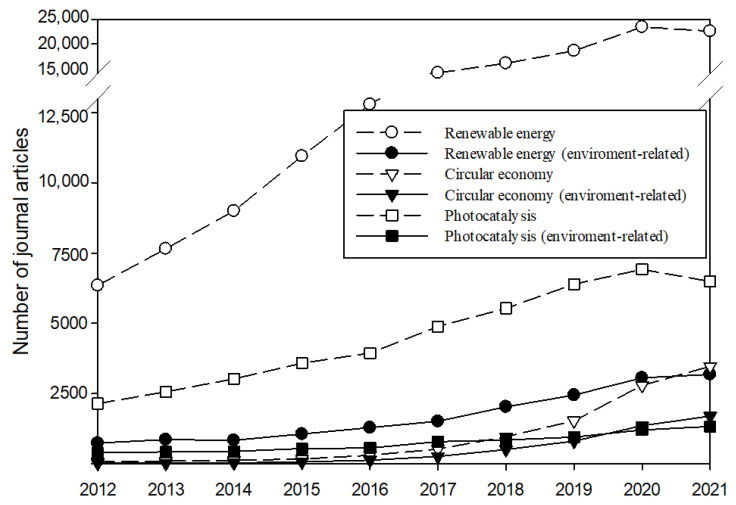
SCI journals focusing on the circular economy, renewable energy, and photocatalysis. Environment-related represents the articles associated with the categories of environmental engineering, environmental science, or environmental studies.

A circular economy promises a comprehensive solution to resource efficiency given the concern of non-renewable energy scarcity. Besides raw materials, energy sources such as renewables are becoming increasingly viable alternatives to fossil fuels. These factors have combined to increase the research activity into the circular economy and renewable resources, as shown in Figure 1. To date, it is estimated that more than 10,000 studies associated with renewables have been reported, as part of these have focused on their applications in the fields of pollution prevention and control. For instance, ethanol is currently known as an alternative fuel from agricultural, industrial and urban residues [1,2]. Electrochemical technologies replace or reduce hazardous materials used in conventional chemical treatment processes [3,4]. Among these discussions, photocatalysis demands an approach associated with the intermittent nature of sunlight, which is considered a renewable energy source, or the assistance of ultraviolet (UV) light. The recognition of the interesting fact, including chemical reactions enabled and/or powered by the energy delivered by photons, relatively higher reaction rates with lower energy requirement, and repeatability of catalysts, has inspired generations of scientists to develop technologies more efficient and less costly to meet the needs for environmental treatment and remediation.

Titanium dioxide (TiO_2_) is one of the typical catalysts that has been used for pollution control [5]. Graphene is a two-dimensional carbon allotrope with premium thermal and electrical properties [6,7]. As combining TiO_2_ with graphene has shown promising results in the research of photocatalysis, our goal in writing this review is to provide a broad overview of this field. We aim to offer a historical account of the development and uses of TiO_2_ and graphene family nanomaterials (GFN) for photocatalysis. Most importantly of all, we hope to unify the discussion of these two materials and provide readers an opportunity to focus on the critical scientific merits of combining TiO_2_ and GFN for enhanced photocatalysis. The article is organized in the following ways. We start with the basic principles that govern photocatalysis and then move on to introduce the historical views of TiO_2_ and GFNs. Their emergences and preparation methods are summarized, followed by a discussion on their physical and electrochemical properties. Afterward, the review examines the integration of TiO_2_ and GFNs as an emerging photocatalyst for the treatment of different water and air pollutants that have been reported in the studies. At the end of this review, we present our perspectives on where the research field of this integrated photocatalysis could be headed.

## 2. TiO_2_

### 2.1. Background

TiO_2_ is a naturally occurring oxide of titanium with structural stability and corrosion resistance [8,9,10]. Although TiO_2_ is typically considered to be of low toxicity, the development of TiO_2_ nanotechnologies has resulted in increased human and environmental exposure, putting TiO_2_ nanoparticles under toxicological scrutiny. Evidence in experimental animals for the carcinogenicity of TiO_2_ has been reported [11]. The International Agency for Research on Cancer (IARC) has indicated that TiO_2_ is possibly carcinogenic to humans [12]. Fujishima and Honda (1972) first discovered UV-light induced electrocatalysis for the splitting of water by using TiO_2_ as a photoanode in an electrochemical cell [13]. Frank and Bard (1977) reported the heterogeneous photocatalytic oxidation of cyanide in water using TiO_2_ powder [14,15]. Since then, photocatalysis using TiO_2_ has achieved a burst of interest to researchers due to the potential implications in the fields of environmental treatment and pollution control [16,17]. TiO_2_ is commonly present in the structures of anatase, brookite, and rutile [18]. Although rutile is the most abundant form of TiO_2_ with thermal stability [19], anatase TiO_2_ has improved photosensitive properties due to its excellent charge-carrier mobility and a greater number of surface hydroxyl groups [20]. To date, TiO_2_-based photocatalysis has become a viable technology for various purposes, including treatment of a wide range of environmental pollutants and eco-friendly green processes of organic synthesis.

### 2.2. Photocatalysis

**Figure 2:**
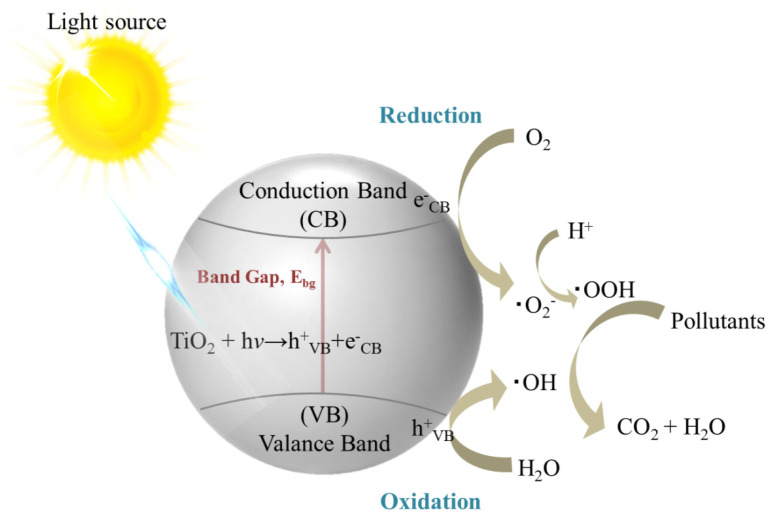
Scheme illustration of a particulate photocatalyst for the mineralization of pollutants in the environment.

Photocatalysis occurs by utilizing light and semiconductors as the substrate [21], as illustrated in Figure 2. The substrate absorbs light and alters the rate of a chemical reaction. In this phenomenon, when a substrate adsorbs photons with the energy exceeding the bandgap energy, an electron-hole (e^−^−h^+^) pair is formed by exciting electrons from the valence band to the conduction band. The existence of the valence band holes (h_VB_^+^) and conduction band electrons (e_CB_^−^) is typically transient and rapidly removed by recombination with heat or light emission. For certain materials, namely photocatalysts, the lifetime of the e^−^−h^+^ pair is extended, allowing a fraction of the e^−^−h^+^ pairs to migrate through the substrate to the surface, performing redox reactions in the surrounding medium [22,23]. The h_VB_^+^ can oxidize water and hydroxyl anions to generate hydroxyl radicals (OH), while dissolved oxygen can be reduced by the e_CB_^−^, leading to the formation of superoxide radical anions (O_2_^−^) or hydroperoxyl radicals (OOH) with further protonation [24]. These strong oxidizing radical species allow the degradation or mineralization of pollutants in the environment upon the exposure of a photocatalyst to light.

### 2.3. Synthesis

The structural, electronic, and optical properties of TiO_2_ are affected by using materials with different sizes, shapes, or phases for synthesis. However, the method used for synthesis is the key to determining the TiO_2_ product characteristics. To date, TiO_2_ is synthesized by using methods including the sol-gel method, micelle and inverse micelle method, sol method, hydrothermal method, solvothermal method, direct oxidation method, chemical vapor deposition, physical vapor deposition, electrodeposition, sonochemical method, flame pyrolysis, and microwave method [25]. Table 1 lists the methods that are widely used and provides more detailed descriptions. The sol-gel method is one of the most commonly used approaches. This approach produces TiO_2_ particles with high crystallinity, limited agglomeration, as well as good size distribution and dispersity. Additionally, the formation of rutile can be controlled by temperature adjustment in this procedure, as anatase materials are effectively obtained at low temperatures.

### 2.4. Properties between Different Polymorphs

TiO_2_ is typically recognized to occur in three different polymorphs, including rutile, anatase, and brookite. The latter is rarely used as a catalyst because it is difficult to synthesize. The photocatalytic activities of rutile and anatase TiO_2_ are dependent upon the crystal structure, size distribution, surface area, pore structure, etc. Despite its low bandgap (Table 2), the lower photocatalytic activity of rutile TiO_2_ is correlated to the intrinsic recombination of photogenerated e^−^−h^+^ pairs [35]. It has been reported that the bulk transport of excitons to the surface contributed to the different photocatalytic activities between the rutile and anatase TiO_2_, as charger carriers excited deeper in the bulk contribute to more efficient photocatalysis in anatase than in rutile [36]. Furthermore, compared to the rutile structure, the photocatalytic activity of anatase TiO_2_ is improved by its smaller particle size [37], higher surface area [38], and more importantly, higher surface-adsorbed hydroxyl radicals and the slower so-called photoinduced charge-carrier recombination in anatase relative to rutile [39,40]. The increased lifetime of the e--h+ pair can predominate over the charge-hole recombination process. The lower effective mass of the photogenerated charge carrier can increase the mobility of electron transfer. These characteristics enhance the photocatalytic activity of the crystalize anatase, thereby making it the most active catalyst compared to rutile and brookite. Table 2 compares the properties imperative to TiO_2_ in its anatase, rutile, and brookite crystalline phases.

Although the discussion above promotes the use of the anatase as the catalyst of preference compared to rutile, a larger intrinsic bandgap of anatase TiO_2_ (3.2 and 3.0 eV for anatase and rutile structures, respectively) only allows a smaller portion of the solar spectrum in the UV light region to be adsorbed, thereby negatively affecting the applicability of this technology. One solution is the doping of different ions that contributes to the improved activities of TiO_2_ in different ways. For example, doping with Fe or Zn improves the conductivity of TiO_2_ and the mobility of charge carriers, slowing recombination and more efficiently separating photogenerated electrons and holes [49]. Recently, the non-metal doping of C has been extensively investigated due to its improved response to visible light and high photostability. The replacement of O in the TiO_2_ lattice with C narrows the bandgap and promotes the adsorption of the main region of the solar spectrum. Furthermore, impurity states formed near the valence band edge along with C-doping can act as shallow traps and extend the occurrence of photogenerated electron-hole pairs [49,50], as graphene represents one emerging material increasingly used for this purpose.

## 3. Graphene Family Nanomaterials (GFN)

### 3.1. Graphene and Its Derivatives

**Figure 3:**
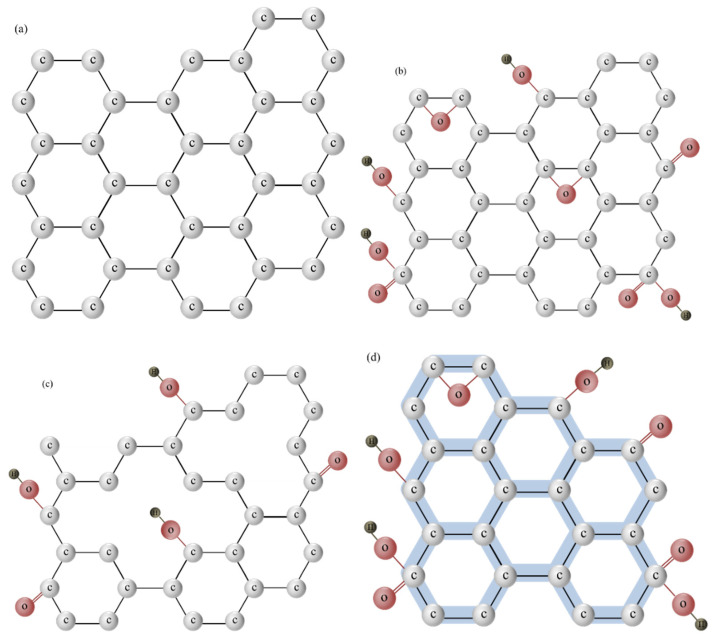
Chemical structures of (**a**) graphene; (**b**) graphene oxide (GO); (**c**) reduced GO (rGO); and (**d**) graphene quantum dots (GQDs).

Since its successful extraction from graphite in 2004 [51], research with this material stems from its exceptional electrical, mechanical, and optical properties and the potential applications employing these properties. GFN includes graphene oxide (GO), reduced GO (rGO), and graphene quantum dots (GQDs) [52], as illustrated in Figure 3. Graphene is a two-dimensional carbon allotrope, as the sp^2^ hybridization results in the extreme of such properties including high conductivity, remarkable optical features, and mechanical strength along two dimensions [51,53]. GO is the sheet of a defective sp^2^ carbon network that incorporates oxygenated groups, including hydroxyl, epoxy, carbonyl, and carboxyl groups, at the interior and on the edge [7,54]. These groups tend to change the surface properties of GO from being hydrophobic to hydrophilic. By reducing the oxygen content and generating different defects in GO, a material with intermediate features between pristine graphene and GO, namely reduced or partially rGO, is produced [55,56]. Different methodologies applied for GO reduction affect the types and numbers of defects and thereby the chemical properties of the final product. GQDs that contain both sp^2^ and sp^3^ hybridizations are separated from the single to several layers of graphene sheets to several nm in lateral size [57]. The features of GQDs include the size-dependent optical band gap, high electron mobility, excellent solubility, and easy functionalization.

### 3.2. Synthesis

Particular emphasis is directed toward the effects of different synthesis methods on the properties of GFN products and the characterization imperative to determine the quality of the synthesis [53,58]. Different synthesis methods and operational factors are known to change the distances between the layers (d-spacing), layer number, stacking order, and structure completeness, which further influences the quality of GFNs. For example, Wu et al. have revealed that the number of graphene layers was effectively tuned by selecting suitable starting materials in the chemical exfoliation method [59]. Artificial graphite, flake graphite powder, Kish graphite, and natural flake graphite were used as starting materials to produce single-layer, single- and double-layer, double- and triple-layer, and few-layer (4–10 layers) graphene final products, respectively. Table 3 discusses the GFNs, including graphene, GO, rGO, and GQD, prepared by different synthesis methods and the associated pros and cons.

**Figure 4:**
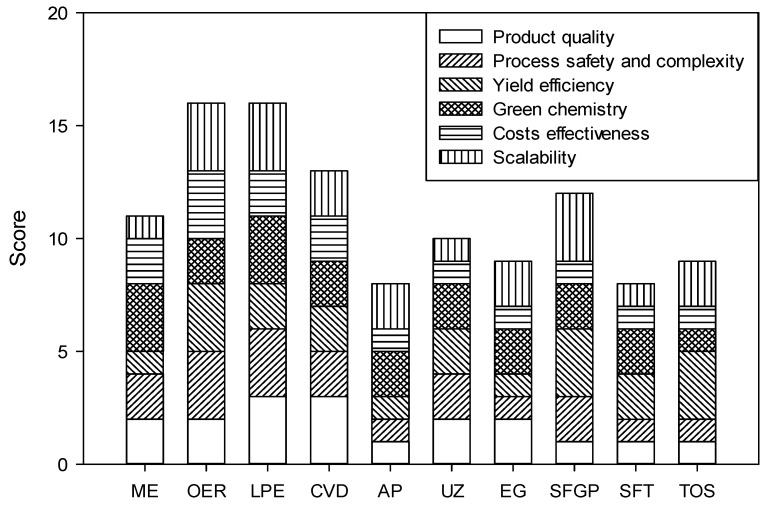
Score evaluation of different methods for graphene synthesis (ME, OER, LPE, CVD, AP, UZ, EG, SFGP, SFT, and TOS represent mechanical exfoliation, oxidative exfoliation-reduction, liquid-phase exfoliation, chemical vapor deposition, arc plasmas, unzipping of carbon nanotubes, epitaxial graphene growth, substrate-free gas-phase synthesis, soft-hard template approach, and total organic synthesis, respectively) (Scoring system: 1-low, 2-medium, and 3-high) [58].

Graphene is typically prepared by using mechanical exfoliation, oxidative exfoliation-reduction (OER), liquid-phase exfoliation (LPE), and chemical vapor deposition, as listed in Table 3. Other emerging methods include arc plasmas [60], unzipping of carbon nanotubes [61], epitaxial graphene growth [62], substrate-free gas-phase synthesis (SFGP) [63], the soft-hard template approach [64], and total organic synthesis [65]. Lee et al. [58] evaluated aspects of product quality, process safety and complexity, yield efficiency, environmental impacts, cost-effectiveness, and scalability among different approaches for graphene synthesis (Figure 4). The popularity of the OER and LPE are explained by their relatively higher scores in each category of comparison.

The synthetic methods of GO were continuously modified in recent decades. The methods that are widely used and frequently discussed include the Brodie method [66], Staudenmaier method [67], Hofmann method [68], and Hummers method [69], as listed in Table 3. The Hummers is typically recognized as one popular method for its efficiency, safety, effective oxidation and crystallinity, and scalable production of large-area and high-quality products. Recently modified Hummers approaches that are more environmentally friendly have emerged as one of the most popular methods for GO production for different purposes. For example, hazardous chemicals such as NaNO_3_ used in conventional Hummers methods that form toxic NO_2_/N_2_O_4_ gases were replaced without a yield decrease in an improved Hummers method [70,71]. Zaaba et al. improved the method by carrying it out at room temperature and without NaNO_3_ [72]. The chemical recipe of the Hummers method was adjusted (e.g., the increase of KMnO_4_ used and change of the H_2_SO_4_/H_3_PO_4_ mixing ratio) to enhance the efficiency of the oxidation process [73]. A different oxidant, K_2_FeO_4_, was studied for its potential to reduce the formation of toxic gases, to enable the recycling of sulfuric acid, and to increase the reaction efficiency [74].

rGO is typically processed by chemical, thermal, and other methods [75,76]. Chemical reduction is commonly used given its merits of fine product quality and scalable production [77,78,79,80]. Thermal reduction is another method for rGO production. These processes are straightforward and cost-effective. However, the needs of certain hazardous reductants or capital costs and energy in chemical and thermal reduction, respectively, resulted in the rise of other emerging methods such as electrochemical reduction [81], microwave and thermal reduction [82]. These technologies provide alternatives with high yield efficiencies, fine product qualities, and the potential for green chemistry.

GQDs are one of a few layers of graphene with a size smaller than 100 nm [83]. GQDs exploit the intrinsic characteristics of graphene nanomaterials and increase their applications with their enhanced and tunable photoluminescence, unique photo-induced redox properties, and high biocompatibility [84,85]. The preparation methods of GQDs include the “top-down” and “bottom-up” methods. The “top-down” methods, which mainly prepare GQDs by chemically, electrochemically, or physically cutting the crystallites of graphene, include hydrothermal synthesis [86], the solvent thermal method [87], chemical oxidation [88], electrochemical exfoliation [89,90,91,92], electron beam lithography [93], the microwave-assisted method [94], and ultra-sonication exfoliation [95,96]. The “bottom-up” methods offer new strategies to fabricate GQDs by pyrolysis of small organic compounds or by chemical fusion of small aromatic compounds. These methods include the soft template method [97], acid and solvent-free synthesis [98], and metal catalysis [99]. Considering the need for hours to develop low-dimensional GQD, the manufacturing of GQDs for industrial-scale applications is still being investigated and increasingly discussed.

### 3.3. Properties

Table 4 lists some properties of GFNs that have been reported in the studies. Graphene has aroused wide attention because of its unique electronic, optical, thermal, and mechanical properties, as the properties of derivatives were known to be changed by different functional groups, structural defects, and stacking layers and sizes. Graphene displays ultrahigh mobility of electrons (e.g., 15,000 cm^2^ v^−2^ s^−1^) which depends weakly on temperature, remarkable mechanical strength (Young’s modulus of 1.0 TPa and fracture strength of 130 GPa), high-frequency optical conductivity from the infrared through the visible range of the spectrum, high thermal conductivity (~4000 Wm^−1^ K^−1^), and the capability of easily converting electrical currents to heat [53].

Although the conjugated regions of GO that are partially destructed by the oxygen-containing functional groups negatively affected its electrical mobility and mechanical strength (the average elastic modulus was 32 GPa, while the highest fracture strength was 120 MPa), GO is stable in water, and this property has provided opportunities for possible applications in solutions [100,101]. The introduction of chemicals such as divalent polyallylamine or metal ions that cross-link between GO layers has improved the mechanical properties of GO [53,102].

**Table 4:** Properties of GFNs that have been reported in studies.

Properties	Graphene	GO	rGO	GQD
Functional group	No functional group	Epoxy, carboxyl, hydroxyl, and carboxyl	Epoxy, carboxyl, and hydroxyl	Epoxy, carbonyl, hydroxyl, and carboxyl
Nature	Hydrophobic	Hydrophilic	Hydrophilic	-
C:O ratio	No oxygen	2-4	8-246	3
d-spacing (nm)	0.335	0.737	0.368	0.381
Surface area (m^2^/g)	2600	487	466	-
Electron mobility (cm^2^V/s)	10,000–50,000	Insulator	0.05–200	-
Resistance (Ω)	7200	0.514±0.236	2.01 ± 1.6	-
Optics	2.3% absorption(visible light)	-	~20% adsorption (400–1800 nm)	-
Thermal conductivity (W/m·K)	~5000	2.94	61.8	-
Zeta potential (mV)	-	−33~−21.46	−23.5~−26.5	8
Young’s modulus	1	0.2	0.25	-
Reference	[79,103,104,105,106,107,108]	[103,104,109,110,111,112]	[77,103,104,110,113,114,115]	[103,104,113,116,117]

As a form of GO that is reduced to destruct the conjugates and to form defects, the structural flexibility (e.g., higher stiffness and tensile strength) and excellent conductivity of rGO have been examined. Sun et al. have reported that adding 0.30 wt% rGO increased the yield strength and ultimate tensile strength of an rGO/Al composite by 15.6% and 11.7% compared with the bare Al material, respectively [118]. The excellent electrochemical properties of rGO-containing metal oxides has allowed them to be suitable candidates for anode materials in battery applications [119].

Because of the quantum confinement and edge effects, GQDs revealed superior luminescence properties, chemical stability, and biocompatibility [120]. There has been much interest in the use of GQDs for applications in microelectronic, sensing, and biomedical technologies [113]. Overall, composites based on graphene or rGO are of increasing interest as the materials for the synthesis of photocatalysts since they have suitable physicochemical and optical properties, such as high specific surface areas, superior electron mobility, and excellent light transmissivity.

## 4. GFN-TiO_2_

### 4.1. Synthesis

Many materials and methods can be used to synthesize TiO_2_-containing composites. It has been reported that these composites can be produced in many different forms, such as nanoparticles [121,122,123,124], nanofibers [125,126,127], and nanosheets [128,129]. The forms affect the physicochemical properties of these composites, such as the specific surface areas, influencing their photocatalytic activities. For example, the synthesis of TiO_2_-containing nanowires [130,131,132,133,134], nanorods [135,136,137,138], and nanotubes [139,140,141,142] with high specific areas that are associated with their improved efficiencies have been revealed. Table 5 lists the selected physicochemical properties of TiO_2_-containing composites prepared in different dimensions. Besides the forms of the catalysts, the materials added in the synthesis of composites are another key. Among various materials, including carbonaceous materials and metal oxides, that are commonly used to enhance their photocatalytic performance [143,144], GFNs have aroused substantial attention recently due to their unique characteristics described above. Table 6 lists the methods that have been reported for the synthesis of GFN-TiO_2_. These methods include ion implantation, sintering at high temperatures, plasma processes, the hydrothermal method, the sol-gel method, hydrolysis, chemical modification, and low-temperature carbonization [32]. The hydrothermal method is the most frequently used method, given the advantages comprising the adjustable crystal form, GFN content, and variable reduction level of an rGO-TiO_2_ [145]. This method is known to avoid the high-temperature destruction of carbonaceous structures and successfully preserve stable and complete crystal forms.

### 4.2. Characterization

Different approaches have been used to study the different surface characteristics and chemical structures of GFN-TiO_2_ (Table 7). Scanning electron microscopy (SEM) [151,163,164,165,166], transmission electron microscopy (TEM) [163,165,166], and atomic force microscopy (AFM) [164] are typically used for morphological observation. The results indicated that GFN was well embedded or covered by TiO_2_. The composites with lower GFN ratios tended to aggregate, forming large spherical-shaped particles [151,163,164,165,166]. Adding graphene increased and then decreased the crystallite size of composites. The initial augmentation was caused by accelerating the crystallization of TiO_2_. Excess H_2_O by the dispersion of graphene promoted the hydrolysis of titanium isopropoxide. Continuously increasing the graphene content enhanced incorporation between the nucleation centers, delaying crystallization and decreasing the crystallite size [151,163,164,165,166]. Composites could exhibit non-spherical structures, such as platelet- or flower-like morphology with elevated GFN ratios [151,163,164,165,166,167]. The TEM studies indicated that GFN-TiO_2_ exhibited two-dimensional structures [163,165,166,167]. An AFM study showed a significant increase in the thickness when excess graphene was added during composite preparation [164].

The chemical constitutions of GFN-TiO_2_ were investigated by using Fourier transform infrared spectrometry (FTIR) [151,165,168], X-ray photoelectron spectroscopy (XPS) [163,164], X-ray diffraction (XRD) [151,163,164,165,166,168], Raman spectrometry [163,164,165], and electron paramagnetic resonance (EPR) [166]. The FTIR results showed that the peak at 400–900 cm^−1^ was broadened or shifted due to the presence of Ti-O-C in the Ti-O-Ti adsorption peak. The original peaks of carbonyl (C=O, 1700 cm^−1^) and epoxy (C-O, 1230 cm^−1^) groups of GO became negligible in the results of GFN-TiO_2_ [151,165,168]. The XPS studies observed the bands of 463.2 and 458.5 eV in GFN-TiO_2_, indicating a chemical state of Ti^4+^ (TiO_2_) in GFN-TiO_2_ [163,164]. The identification of the peaks associated with Ti and GFN indicated the presence of Ti-C, O=C-O-Ti, and C-O-Ti in TiO_2_-GFNs, as the C1s spectrum showed peaks attributed to C=C/C-C, epoxy (C-O)/hydroxyl (C-OH), and carboxyl groups (C(=O)OH). [163,164]. The XRD studies have revealed the peak areas of anatase (25.3°) and a few rutile phases (27.4°), indicating TiO_2_ was well mixed with GFN with limited phase changes [151,163,164,165,166,168]. The Raman spectra of GFN-TiO_2_ exhibited bands of E_g(1)_ (149 cm^−1^), B_1g(1)_ (395 cm^−1^), A_1g_+B_1g(2)_ (517 cm^−1^), and E_g(2)_ (640 cm^−1^), attributable to the symmetric stretching and symmetric/asymmetric bending vibrations of the O-Ti-O group. The spectra also exhibited D (1384 cm^−1^) and G bands (1596 cm^−1^) of GFN, as the D/G intensity ratio was higher than that of GFN [163,164,165]. The EPR study showed increasing intensities of the hydroxyl and superoxide radicals by increasing the ratio of GFN to TiO_2_ [166].

Physicochemical properties including the surface charge, thermal stability, surface area, pore size, and pore volume of TiO_2_-GFN have been investigated by Zeta potential analysis [164,167], thermal gravity analysis (TGA) [164], and Brunauer–Emmett–Teller (BET) analysis [151,163,164,165,168], respectively. The nucleation of TiO_2_ on GFN masked the functional groups on the surface and lowered the zeta potential of GFN-TiO_2_ [164,167]. The TGA study showed a better heat resistance of GFN-TiO_2_, as TiO_2_ stabilized GO by the interaction between oxygen-containing groups of GFN and TiO_2_ [164]. Most studies have indicated a higher surface area of GFN-TiO_2_ compared to that of TiO_2_ [151,163,164,165,168], whereas an opposite trend has also been reported in a few studies [151,163,164,165,168]. GFN-TiO_2_ typically exhibited mesopore size distribution with averages near 10 nm [151,163,164,165,168].

Potentiostat, photoluminescence (PL), and ultraviolet-visible spectroscopy (UV-Vis) are useful tools to investigate the optical characteristics of GFN-TiO_2_ [151,164,165,166,168]. A study has reported that an optimal ratio of GFN to TiO_2_ increased the current density of GFN-TiO_2_, because the two-dimensional conjugation structure of GFN accepted and transported the excited electron from TiO_2_ [168]. Pallotti et al. used photoluminescence (PL) spectroscopy for real-time analysis to trace the time dynamics of the photoreduction of GO [169]. It was found in real-time that the photocatalysis induced by the presence of TiO_2_ contributed to GO photoreduction. By adding GFN into TiO_2_, the absorption edge of GFN-TiO_2_ displayed an increase in wavelength (known as redshift) that indicated a bandgap narrowing. Its light absorption intensity in the UV region was also increased [151,164,165,166,168]. Table 8 lists some examples of TiO_2_-GFN prepared for photocatalysis and battery storage.

### 4.3. Photocatalysis Enhancement

**Figure 5:**
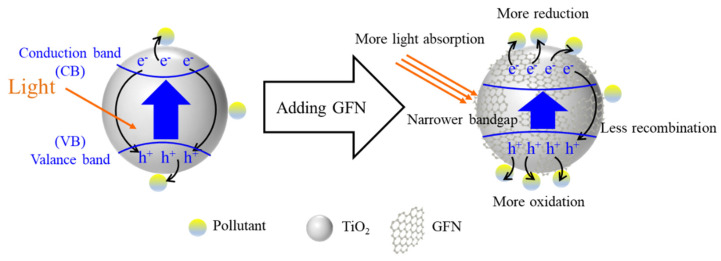
Scheme illustration of enhanced photocatalysis activity of GFN-TiO_2._

Studies have demonstrated the enhanced photocatalysis activity of GFN-TiO_2_, as illustrated in Figure 5. An optimal graphene addition content (e.g., 0.05 wt%) showed photocatalytic activity higher than that of pure TiO_2_ by a factor of 1.7 [163]. The excellent acceptance and transport of electrons by graphene reduced the recombination of charge carriers during photocatalysis. It has been indicated that the excellent conductivity of GFN suppressed the recombination of e^−^−h^+^ pairs, enhancing radical formation and pollutant degradation [151,163,164,165,166,168]. The formation of the Ti-O-C bond of GFN-TiO_2_ effectively reduced the bandgap energy (e.g., 2.66–3.18 eV) [151,166,168]. Compared to pure TiO_2_, GFN-TiO_2_ was more efficient to absorb photons for the generation of e^−^−h^+^ pair due to the shift of the absorption edge toward the visible region [173].

## 5. Photocatalytic Removal of Pollutants

### 5.1. Water-Phase Pollutants

GFN-TiO_2_ has been used for the photocatalytic removal of inorganic, organic, and biological pollutions in the water phase (Table 9). The photocatalytic reduction of inorganic pollutants such as metal ions was one example. Jiang et al. investigated the reduction of Cr(VI) to Cr(III) in water by using GFN-TiO_2_ [164]. The reduction rate constant was 0.0691 min^−1^, exceeding that of using pure TiO_2_ (0.0174 min^−1^) by a factor of 3.9. In another Cr(VI) removal study, the Cr(VI) concentration was adsorbed (~55%) by using TiO_2_-GO for 1 h, and with UV irradiation, nearly all Cr(VI) concentration was reduced in 7 h [174]. In the same system using TiO_2_ with UV irradiation, the Cr(VI) concentrations were limitedly adsorbed (23%) and reduced (30%).

Graphene-TiO_2_ has been frequently investigated for its potentials for photocatalytic degradation of organic pollutants. Homolytic cleavage is typically the first chemical step in photodegradation. Free radicals are formed in this step and rapidly react with any oxygen present in the system. Li et al. investigated the photocatalytic activity of graphene-TiO_2_ towards representative aqueous persistent organic pollutants (POPs) [170]. The POPs included rhodamine B, norfloxacin, and aldicarb. The presence of graphene-TiO_2_ significantly enhanced the removal of these POPs. While the compound concentrations were negligibly changed during pure photolysis, the presence of GFN-TiO_2_ (0.86% *w*/*w* of graphene) resulted in 79.7% and 86.2% of total organic carbon (TOC) removal in the experiments of rhodamine B and norfloxacin, respectively, after 10 h of simulated sunlight irradiation (λ > 320 nm). Only 36.8% of TOC removal was observed in the aldicarb experiment after 25 h of visible light irradiation (λ > 400 nm). Zhang et al. investigated photodegradation of methylene blue by using TiO_2_, carbon nanotube (CNT)-TiO_2_, and graphene-TiO_2_ as photocatalysts [175]. In 1 h of UV irradiation, the removal efficiency of graphene-TiO_2_ (85%) was significantly higher than TiO_2_ (25%) and CNT-TiO_2_ (71%). Using visible light reduced the performance of TiO_2_ by a factor of 2, whereas the removal efficiency of graphene-TiO_2_ (65%) was less affected.

GO represents another material that can work well with TiO_2_, forming an efficient photocatalyst. Perera et al. compared the photodegradation of malachite green by using TiO_2_, GO, and GO-TiO_2_ [145]. Pseudo-first-order reactions were found when TiO_2_ and GO-TiO_2_ were used as catalysts. The rate constant of GO-TiO_2_ (0.0674min^−1^) exceeded that of TiO_2_ (0.0281 min^−1^) by a factor of 3. No photodegradation of malachite green occurred in the presence of GO. Another study investigated the photodegradation of rhodamine B by using three different nanosphere catalysts (amine-modified TiO_2_–SiO_2_, graphene-TiO_2_, and GO-TiO_2_–SiO_2_) [176]. In 1.5 h of irradiation, the removal efficiencies of graphene-TiO_2_ (91%) and GO-TiO_2_–SiO_2_ (71%) were significantly higher than that of amine-modified TiO_2_–SiO_2_ (65%), indicating the synergistic effect between graphene or GO and TiO_2_ for the enhanced catalysis activity.

The use of rGO-TiO_2_ for the enhanced photocatalytic degradation of organic pollutants has also been demonstrated. Increasing the rGO content (from 0 to 1% *w*/*w*) in rGO-TiO_2_ enhanced the photocatalytic decomposition of phenol (the 1st-order rate constant was increased from 0.0039 to 0.0048 min^−1^) [177]. rGO-TiO_2_ exhibited fine photocatalytic performance after 5 cycles; however, a high rGO content (e.g., 5% *w*/*w*) potentially shielded the catalyst surface from light absorption, reducing the photocatalytic activity. Ng et al. investigated the removal of 2,4-dichlorophenolyxacetic acid (2,4-D), a commonly used herbicide, by photocatalytic reduction using TiO_2_ and rGO-TiO_2_ [178]. The pseudo-first-order rate constants of using TiO_2_ and rGO-TiO_2_ were 0.002 and 0.008 min^−1^, respectively. Adding rGO increased the response of the photocurrent by a factor of 2 and the availability of 2,4-D on the surface of rGO-TiO_2_, improving the whole photocatalytic reaction by a factor of 4.

Photocatalysis is capable of being adopted for use in many applications for disinfection in water matrices. Adding graphene in Ag_3_PO_4_-TiO_2_ effectively improved the synergistic photocatalytic disinfection of *E. coli*, *S.aureus*, *S.typhi*, *P. aeruginosa*, *B. subtilis*, and *B. pumilus* [180]. Fernández-Ibáñez et al. have reported effective solar photocatalytic disinfection of *E. coli* and *F. solani spores* by using rGO–TiO_2_. The presence of rGO significantly enhanced the performance of photocatalytic disinfection of *E. coli*. Increasing rGO–TiO_2_ from 0 to 500 mg/L accelerated the inactivation of *E. coli* (10^6^ colony-forming units (CFU)/mL) from more than 100 to 10 min and reduced the solar UV dosage needed from 123 to 11 kJ/m^2^. Although both rGO-TiO_2_ and pure TiO_2_ exhibited excellent disinfection of *F. solani spores*, rGO significantly reduced the solar energy required from 336.2 to 42.1 kJ/m^2^ [179]. A certain ratio between rGO and TiO_2_ significantly enhanced the photocatalytic disinfection under UV and solar irradiation [182]. Another study has also demonstrated that GO, which effectively separated photo-generated e^−^−h^+^ pairs for more ▪OH production, improved the photocatalytic disinfection of *E. coli*. In 30 min, the disinfection efficiencies of using pure TiO_2_, GO, GO-TiO_2_ were 39.27%, 73.82%, 99.60%, respectively [181]. More detailed information concerning the removal of different inorganic, organic, and biological pollutants by using GFN-TiO_2_ is available in Table 8.

### 5.2. Air-Phase Pollutants

Similar to the removal of pollutants in the water phase, GFN-TiO_2_ has been adopted for use in removing a wide range of air pollutants. Shorter contact times and the complexity of the heterogeneous photocatalytic reactions (e.g., photon absorbance and radical reactions) between pollutants and catalyst surfaces represent two typical challenges in this field [151].

In the aspect of inorganic removal, the treatment efficiencies of gaseous NOx (from NO_(g)_ to NO_2(g)_ to NO_3_^−^_(s)_) by using pure TiO_2_, graphene-TiO_2_, and rGO-TiO_2_ were compared [165]. An appreciable level of GFN (e.g., 0.01–0.1% graphene or rGO) in TiO_2_ improved the removal of NOx under UV and visible light. The NOx removal efficiencies were 25.45%, 26.26–35.40%, and 39.38–42.86% by using TiO_2_, graphene-TiO_2_, and rGO-TiO_2_ under UV light, respectively, while under visible light the removal efficiencies using TiO_2_, graphene-TiO_2_, and rGO-TiO_2_ were 9.35%, 15.20–22.75%, and 19.88–22.34%, respectively. Giampiccolo et al. prepared graphene-TiO_2_ by using the sol-gel method for electrochemical sensing and photocatalytic degradation of NOx in the air [183]. Interestingly, the performances of graphene-TiO_2_ prepared by using the same method but with different step orders were compared (adding graphene to the reaction before initiating the sol-gel reaction followed by annealing (GTiO_2_S) and adding graphene to TiO_2_ which had already been annealed (GTiO_2_M)). The addition of graphene significantly improved the performance of the catalysts under solar irradiation (280–780 nm) (e.g., the pseudo-first-order rate constants of NOx removal by GTiO_2_S, GTiO_2_M, and TiO_2_ were 6.7, 5.6, and 4.3/min, respectively.). The thermal treatment helped synthesize graphene and TiO_2_ in more intimate contact and improved the exhibition. Besides NOx, photodegradation of CO by using GO-TiO_2_, which was functionalized by attaching a cobalt (Co)-imidazole (Im) complex on GO, was investigated [109]. The results revealed that the bandgaps of this functionalized GO-TiO_2_ (with Co and Im), GO-TiO_2_, and pure TiO_2_ were 2.78, 2.96, and 3.10 eV, respectively. The removal efficiencies of CO and NOx were improved from 10% to 46% and from 16% to 51% when the catalyst was changed from TiO_2_ to the functionalized GO-TiO_2_, respectively. Xu et al. added graphene into TiO_2_ to enhance the photocatalytic CO_2_ conversion to chemical fuels [184]. The addition of graphene inhibited the recombination of e^−^−h^+^ pairs and raised the surface temperature, improving the CO_2_ conversion efficiency. The conversion rates of CO_2_ to CH_4_ and CO by using graphene-TiO_2_ were higher than those using TiO_2_ by factors of 5.1 and 2.8, respectively.

Studies have demonstrated the photocatalytic degradation of organic pollutants in the air phase by using GFN-TiO_2_. Zang et al. have reported that adding graphene into TiO_2_ with a specific ratio (e.g., 0.5% *w*/*w*) exhibited a synergetic effect on the UV light photodegradation of benzene (the mineralization rates of GFN-TiO_2_ and TiO_2_ were 76.2% in 10 h and 1.2% in 28 h, respectively). The adsorption of benzene and intermediates during benzene degradation negatively affecting TiO_2_ adsorbing UV light was decreased by the presence of graphene. However, excess graphene could adsorb extra compounds and impact light absorption. Benzene removal was limitedly found when visible light was used [166]. Similarly, in a study that focused on the photocatalytic degradation of acetone in the air, graphene-TiO_2_ exhibited a better activity (the pseudo-1^st^-order rate constant was 10.2 × 10^−3^/min) exceeding that of pure TiO_2_ (5.99 × 10^−3^/min) by a factor of 1.7 and a good reproducibility after three cycles of illumination [163].

Adding other materials to graphene-TiO_2_ has been investigated to further enhance its photocatalytic activity. Photocatalytic degradation of formaldehyde by using graphene-TiO_2_-Fe^3+^ has been reported [168]. Under UV light, both graphene-TiO_2_-Fe^3+^ and graphene-TiO_2_ revealed better performances than pure TiO_2_, as only the photolytic activity of graphene-Fe^3+^-TiO_2_ was better under visible light irradiation. The photocatalyst with a TiO_2_/graphene ratio of 50 and a ratio of Fe^3+^/graphene-TiO_2_ of 0.12% revealed the optimal performance. Nitrogen has been doped into reduced graphene-TiO_2_ to change the polarity of the catalyst and to influence the adsorption and photodegradation of polar acetaldehyde and nonpolar ethylene [185]. Both reduced graphene-TiO_2_ and N-doped reduced graphene-TiO_2_ exhibited higher treatment efficiencies than pure TiO_2_. One explanation was that nitrogen doping improved the polarity of the catalyst, further enhancing the removal efficiency of polar acetaldehyde.

The feasibility of adding GO into TiO_2_ for the photocatalytic degradation of organic pollutants has been reported. A study used GO-TiO_2_ as a photocatalyst to accelerate the degradation of benzene, toluene, ethylbenzene, and xylene (BTEX) in the air [151]. Under UV irradiation, the removal of these compounds by using GO-TiO_2_ was higher than that of using pure TiO_2_ by a factor of 1.2, while GO-TiO_2_ exhibited an excellent treatment efficiency exceeding that of pure TiO_2_ by a factor of 12 under visible light irradiation. GO-TiO_2_ has also been used for the photocatalytic degradation of methyl ethyl ketone in indoor air [171]. The addition of GO in TiO_2_ has improved the removal efficiency from 32.7% to 96.8% under visible light irradiation. Proper humidity (e.g., 40%), flow rate (e.g., 50 mL/min), and pollutant concentration (e.g., 30 ppmv) were the key to optimal performance. Note that the use of nanostructured membranes based on polymeric nanofibers using TiO_2_ and GFNs, including GO, rGO, and few-layer graphene, for the photocatalytic oxidation of gas-phase methanol has been reported. As the photocatalytic activity was greatly changed by the membrane structure and affected by the affinity of GFN to the polymer matrix, rGO exhibited better performance due to its more enhanced electron mobility [186]. Table 10 summarizes the applications of GFN-TiO_2_ for the photodegradation of organic pollutants in the air in these studies.

## 6. Conclusions and Future Work

TiO_2_ has been intensively investigated in early studies given its photocatalytic effects for radical production degrading a wide range of pollutants in the environment. TiO_2_ with an anatase-crystal structure generally exhibited higher photocatalytic activity than rutile TiO_2_. Its intrinsic properties, including the surface area, adsorption capacity, bandgap, and lifetime of the e^−^−h^+^ pair, have provided opportunities for applications under UV light irradiation. However, these properties could be improved to guarantee a wider range of applications, such as those for visible-light or solar irradiation. The advantages conferred by the physical, optical, and electrochemical properties of GFN have contributed to the current variety of GFN-TiO_2_ catalysts that exhibit improved characteristics, such as higher surface areas, more rapid electron transfer, and narrower bandgap. Although the physicochemical properties and photocatalytic activity could be different between GFN-TiO_2_ prepared by different methods, many studies presented in this review have demonstrated that the applications of using GFN-TiO_2_ have greatly improved photocatalytic reactions for the treatment of organic, inorganic, and biological pollutants in water and air phases. GFN-TiO_2_ exhibited better photocatalytic activity than pure TiO_2_ under UV light irradiation, as the improvement is more significant under visible-light irradiation.

Note that the ratio of GFN and TiO_2_ in GFN-TiO_2_ is typically the key to optimizing the photocatalytic reactions in many studies. Excess GFN could increase the opacity of GFN-TiO_2_, limiting the light absorption of TiO_2_ and negatively affecting the formation of e^−^−h^+^ pairs. Besides the type of GFN (e.g., graphene, GO, and rGO), different preparation methods affected the properties of GFN-TiO_2_ products. Recently, studies have turned their attention to green chemistry that could use fewer chemicals or energy for the preparation of GFN and GFN-TiO_2_. Examples include the electrochemical exfoliation of graphene and the UV-assisted photoreduction of GO. The applications of GFN-TiO_2_ for the removal of inorganic pollutants in the water, such as photocatalytic reactions of ammonium and nitrite and inactivation of biological pollutants in the air, were relatively limitedly examined and represent other directions of technological innovation and possible future development in these fields.

## Figures and Tables

**Table 1:** Summary of the methods widely used for the synthesis of TiO_2._

Method	Mechanism	Phase of Formation	Pros and Cons	Reference
Sol-gel	Hydrolysis and condensation of TiCl_4_ or an organometallic compound	Amorphous and rutile	High purity, fine particle sizes, good size distribution, high surface areas, but the ease of agglomeration and long reaction time	[25,26,27,28]
Hydrothermal	Precipitation of TiO_2_ from aqueous solution at elevated temperature and pressure	Anatase and rutile	High crystallinity, low defects, fine particle size, good size distribution, limited agglomeration, control of crystal shape by temperature adjustment, but relatively higher costs	[25,29,30]
Solvothermal	Precipitation of TiO_2_ from organic solution at elevated temperature and pressure	Anatase and rutile	High crystallinity, low defects, suitability for materials unstable at high temperature, but organic solvents needed	[25,31]
Micelle and inverse micelle	Aggregation of TiO_2_ in a liquid colloid	Amorphous	High crystallinity, low defects, fine particle sizes, but relatively high costs and high crystallization temperatures	[25,32]
Flame pyrolysis	Combustion of TiCl_4_ with oxygen; used in industrial processes	Anatase and rutile	Rapid and mass production, but high energy needed and ease of rutile formation	[25,33,34]

**Table 2:** Comparison of different polymorphic forms of TiO_2._

Properties	Anatase	Brookite	Rutile
Crystal structure	Tetragonal	Orthorhombic	Tetragonal
Density (g/cm^3^)	3.79	3.99	4.13
Band gap (eV)	3.2 ^a^	~3.2 ^b^	3.0 ^c^
Light absorption (nm)	<390	-	<415
Dielectric constant	6.04	7.89	6.62
Lattice energy (kJ/mol) ^d^	24.75	18.53	0
Surface enthalpy (J/m^2^) ^e^	1.34	1.66	1.93
Photocatal. activity (mol/h) ^f^	3.5 × 10^−5^	-	1.1 × 10^−5^
Effective electron mass (m_e_*/m_0_) ^g^	0.0948	0.0949	1.4640
Effective hole mass (m_h_*/m_0_) ^g^	0.1995	0.5620	0.4345
Ti-O bond length (Å) ^h^	1.94 (shorter); 1.97 (longer)	1.87–2.04	1.95 (shorter); 1.98 (longer)
O-Ti-O bond angle (degree)	77.7; 92.6	77.0–105	81.2; 90.0

Reference sources: ^a^ [41]; ^b^ [42]; ^c^ [43]; ^d^ [44]; ^e^ [45]; and ^f^ [40]; and ^g^ [46]. The other numbers are sourced from [47,48]. ^h^ Anatase and rutile TiO_2_ have two different interatomic distances, while brookite TiO2 has six different Ti-O bonds with a distance ranging from 1.87 to 2.04 A.

**Table 3:** Comparison of different synthesis methods of GFNs.

	Method	Major Approach	Pros and Cons	Cost
Graphene	Mechanical exfoliation	Micro-mechanical cleavage, sonication, ball milling, and fluid dynamics	Straightforward and eco-friendly processes, fine product qualities, but relatively higher costs and limits of scalable production	High
	Oxidative exfoliation-reduction	Chemical reduction, thermal reduction, and electrochemical reduction	Straightforward processes, cost-effectiveness, scalable production, but possible structural damage due to mal exfoliation, and potential use of hazardous chemicals	Low
	Liquid phase exfoliation	Sonication with proper solvents	Straightforward and eco-friendly processes (solvents recyclable), fine product qualities, scalable production, but parameters (e.g., solvent and ultra-sonication) critical to avoid physical deformation and defects	Moderate
	Chemical vapor deposition (CVD)	Thermal CVD, plasma-enhanced CVD, and thermal decomposition	Highly connected products with low defects and high surface areas, but relatively higher costs, limited yields, and high technical thresholds	Moderate
Graphene oxide	Brodie	Graphite + H_2_CO_3_ (C/O ratio = 2.23)	Adjustable oxidation states, but potentials of long reaction time and production of explosive ClO_2_ and acid fog	Low
	Staudenmaier	Graphite + HNO_3_ (fuming) + H_2_SO_4_ + KClO_3_ (C/O ratio = 2.52)	Adjustable oxidation state, but long reaction time and low temperatures to avoid exothermic reactions	Low
	Hofmann	Graphite + HNO_3_ + H_2_SO_4_ + KClO_3_ (C/O ratio = 2.52)		Low
	Hummers	Graphite+NaNO_3_ +H_2_SO_4_+ KMnO_4_ (C/O ratio = 2.1-2.9)	Safe and fast reactions, but more parameters to control	Low
Reduced graphene oxide	Chemical reduction	Various reductants	Fine product qualities, scalable production, but the potential of using hazardous reductants. Lower product qualities and removal of excess chemicals with the use of green reductants	Low
	Thermal reduction	1000–1100 °C for 30–45 s in the absence of air	Straightforward and eco-friendly processes, cost-effectiveness, but high capital costs and energy needed	Moderate
	Electrochemical reduction	The cathodic potential of 1–1.5 V	Low-defect products, rapid and eco-friendly processes, cost-effectiveness, but lower reduction levels and limited scalable production	Low
	Microwave and photo-reduction	Microwave reaction with visible or UV light	Fast reactions, no chemicals needed, and high yield efficiencies	Low
Graphene quantum dot	Top-down	Hydrothermal synthesis, solvent thermal method, chemical oxidation, electrochemical exfoliation, electron beam lithography, microwave-assisted method, and ultra-sonication exfoliation	Scalable production, but difficulty of effective size control	High
	Bottom-up	Soft template method, acid- and solvent-free synthesis, and metal catalysis	Effective size control, but long reaction time and limited scalable production	High

**Table 5:** Selected physicochemical properties of TiO_2_-containing composites prepared in different dimensions.

Dimension	Structure	Surface Area	Light Absorption Wavelength	Current Density	Reference
0	Nanoparticle (less than 100 nm)	180–250 m^2^/g	Ultraviolet to infrared radiation	Not available	[121,122,123,124]
1	Nanofiber	52–55 m^2^/g	<510 nm	0.06 mA/cm^2^	[125,126,127]
	Nanowire	61.5–92.6 m^2^/g	250–540 nm	1.6 mA/cm^2^	[130,131,132,133,134]
	Nanorod	104.6 m^2^/g	~380 nm	0.8 mA/cm^2^	[135,136,137,138]
	Nanotube	400 m^2^/g	<500 nm	0.02 mA/cm^2^	[139,140,141,142]
2	Nanosheet	31–146 m^2^/g	200–900 nm	0.03 mA/cm^2^	[128,129]
3	Porous film	36.4–70.8 m^2^/g	200–700 nm	18.54 mA/cm^2^	[146,147,148,149]

**Table 6:** The synthesis methods of TiO_2_-GFN composites.

Methods	Crystal Form	GFN Ratio	Pros and Cons	Reference
Ion implantation	Anatase	Not available	Fast production, few interfacial defects, great optical character, but high energy costs	[150]
Colloidal blending process	Anatase or rutile	adjustable	Aging at room temperature and vacuum drying needed	[151,152]
Spark plasma sintering	Rutile	1% *v*/*v*	Fast production, but high energy costs and increased rutile form	[153]
Hydrothermal method	Anatase	adjustable	Adjustable doping ratio, but high pressure needed	[154,155,156]
Sol-gel method	Anatase	48% *w*/*w*	Aging at room temperature, long reaction time, and calcination needed	[157]
Hydrolysis	Anatase	16% *w*/*w*	Great heterogeneous nucleation, but longer reaction time and calcination needed	[158]
UV-assisted photo-reduction	Not available	Not available	Fast production and few collapses during reduction, but extra light source needed	[159,160]
In-situ assembly	Anatase	Not available	No calcination and full anatase formation, but long synthesis time	[161,162]

**Table 7:** Methods and outcomes of characterization of TiO_2_-graphene composites.

Category	Technology	Description	Ref.
Morphology	SEM	Spherical and non-spherical (platelet- or flower-like) shapes were observed with low and high GFN contents, respectively.	[151,163,164,165,166,167]
	TEM	A fine dispersion of TiO_2_ in GFN with low- and nano-dimensions was reported.	[163,165,166,167]
	AFM	The thickness of GFN-TiO_2_ was increased to a scale of μm after preparation.	[164]
Chemical constitution	FTIR	The peak of Ti-O-Ti at 400–900 cm^−1^ was broadened or shifted by the influence of Ti-O-C. The signals of carbonyl and epoxy groups were reduced.	[151,165,168]
	XPS	The formation of C-Ti, O=C-O-Ti, and C-O-Ti bonds in GFN-TiO_2_ was observed.	[163] [164]
	XRD	The signals due to the presence of anatase and rutile were reported.	[151,163,164,165,166,168]
	Raman	The signals of both TiO_2_ and GFN were reported. The D/G intensity ratio of GFN-TiO_2_ was higher than that of GFN.	[163,164,165]
	EPR	The formation of hydroxyl and superoxide radical species was observed in GFN-TiO_2_.	[166]
Physicochemical properties	Zeta potential	The zeta potential of GFN-TiO_2_ ranged between those of GFN and TiO_2_.	[164]
	TGA	The irregular mass loss occurred at high temperatures.	[164]
	BET	The surface area of GFN-TiO_2_ was significantly increased at a certain ratio of GFN to TiO_2_.	[151,163,164,165,168]
	ACM	The current density of GFN-TiO_2_ was significantly increased at a certain ratio of GFN to TiO.	[168]
	PL	The time dynamics of the TiO_2_-induced photoreduction of GO were observed.	[169]
	UV-Vis	A shift to larger wavelengths in the absorption edge was observed, indicating bandgap narrowing.	[151,164,165,166,168]

**Table 8:** Properties of TiO_2_-GFN prepared for photocatalysis and battery storage in various studies.

Materials	Average Size (nm)	Functional Group	Bandgap (eV)	Wavelength (nm)	Surface Area (m^2^/g)	Reference
Graphene-TiO_2_	3.8	C-O, C=O, O=C-O, and O-Ti	NA ^1^	600	176	[170]
Graphene-TiO_2_	~6	C-O and O-C=O	NA	NA	252	[158] ^2^
GO-TiO_2_	NA	C-O, Ti-O-Ti, Ti-O-C, and OH	NA	~800	69.2	[151]
GO-Co-TiO_2_	NA	C-O, C-N, O-C=O	2.77	421	206	[109]
GO-Ti	NA	NA	2.9	~550	68.9	[171]
rGO-TiO_2_	35	NA	NA	~360	212.75	[172]
rGO-TiO_2_	~8	NA	NA	NA	229	[157] ^2^

^1^ NA denotes not available. ^2^ The materials were prepared for battery storage.

**Table 9:** Removal of water-phase pollutants by GFN-TiO_2_ in selected studies.

	Pollutant	Catalyst	Light Source	Removal	Ref.
Inorganic	Cr(VI) (0.2 mM)	GO-TiO_2_ (0.5 g/L)	254 nm, 20 W, UV lamp	90%	[164]
	Cr(VI)(10 mg/L)	GO-TiO_2_ (0.5 g/L)	365 nm, 8 W, UV lamp	99%	[174]
Organic	Methylene blue (0.01 g/L)	Graphene-TiO_2_ (0.75 g/L)	365 nm, 100 W, high-pressure Hg lamp >400 nm, 500W, Xe lamp	85% 65%	[175]
	Rhodamine B (20 mg/L)	Graphene-TiO_2_ (0.1 g/L)	11 W, low-pressure Hg lamp	91%	[176]
	Rhodamine B (20 mg/L) Norfloxacin (20 mg/L) Aldicarb (10.5 mg/L)	Graphene-TiO_2_ (1 g/L)	>400 nm, Xe lamp	79.7% 86.2% 36.8%	[170]
	Malachite green oxalate (13.1 mg/L)	GO-TiO_2_ (0.2 g/L)	450 W, water-cooled Hg lamp	80%	[145]
	Phenol (10 mg/L)	rGO-TiO_2_ (5 g/L)	310-400 nm, UV lamp	Not given	[177]
	2,4-D (15 mM)	rGO-TiO_2_ (film)	<320 nm, 450 W, Xe lamp	~87%	[178]
Biological	*E. coli* (10^6^ CFU/mL), *F. solani spores* (10^3^ CFU/mL)	rGO-TiO_2_ (0.5 g/L)	Sunlight	~100%	[179]
	*E. coli, S.aureus, S.typhi, P. aeruginosa, B. subtilis, B. pumilus* (10^6^ CFU/mL)	Graphene-Ag_3_PO_4_-TiO_2_	>420 nm, 350 W, Xe lamp	~100%	[180]
	*E. coli* (10^5^–10^6^ CFU/mL)	GO-TiO_2_ (0.2 g/L)	Xe lamp	~100%	[181]
	*E. coli* (10^6^ CFU/mL)	rGO-TiO_2_ (18 mg/L)	>285 nm, UV-visible light; >420 nm, visible light	~100%	[182]

**Table 10:** Removal of air-phase pollutants by GFN-TiO_2_ in selected studies.

	Pollutant	Catalyst	Light Source	Humidity or Flow Rate	Removal	Ref.
Inorganic	NOx (1 ppm)	Graphene-TiO_2_ rGO-TiO_2_	15 W, UVA 8 W, visible light	50% humidity, 3 L/min	42% 49%	[165]
	NOx (200 ppb)	Graphene-TiO_2_	280–780 nm, 300 W, solar lamp	1 L/min	77%	[183]
	CO (50 ppm) NOx (1 ppm)	Graphene-TiO_2_	8 W, UV lamp	0.2 L/min	46% 51%	[109]
Organic	Acetone (300 ± 20 ppm)	Graphene-TiO_2_	365 nm, 15 W, UV lamp	1 L/min	~60%	[163]
	Acetaldehyde (500 ppm) Ethylene (50 ppm)	Graphene-TiO_2_	260 W, fluorescent lamp 500 W, Xenon lamp	20 cm^3^/min	~82% ~90%	[185]
	Benzene (250 ppm)	Graphene-TiO_2_	254 nm, 4 W, UV lamp	20 mL/min	6.4%	[166]
	Formaldehyde (3000 ppm)	Graphene-TiO_2_	365 nm, 8 W, black light blue lamp >420 nm, 8 W, fluorescent lamp	Not specified	50.3% 25.5%	[168]
	Methanol (4,000 ppm)	Graphene-TiO_2_ GO-TiO_2_ rGO-TiO_2_	254 nm, 16 W, UV lamp	155 cm^3^/min	80% 99% 99%	[186]
	BTEX (1 ppm)	GO-TiO_2_	400–720 nm, 8 W, daylight lamp	55% humidity, 1 L/min	96%	[151]
	MEKT (30 ppm)	GO-TiO_2_	80 W, Xe lamp	40% humidity, 50 mL/min	96.8%	[171]

## Data Availability

Not applicable.
